# Quantitative Nuclear Magnetic Resonance Spectroscopy with Overhauser Dynamic Nuclear Polarization

**DOI:** 10.1002/cphc.202401052

**Published:** 2025-07-18

**Authors:** Johnnie Phuong, Raphael Kircher, Sarah Mross, Billy Salgado, Hans Hasse, Kerstin Münnemann

**Affiliations:** ^1^ Laboratory of Engineering Thermodynamics (LTD) RPTU Kaiserslautern Erwin‐Schrödinger‐Straße 44 67663 Kaiserslautern Germany; ^2^ Laboratory for Advanced Spin Engineering ‐ Magnetic Resonance (LASE‐MR) RPTU Kaiserslautern Gottlieb‐Daimler‐Straße 76 67663 Kaiserslautern Germany

**Keywords:** ^1^H, ^13^C, benchtop NMR spectrometer, dynamic nuclear polarization, quantitative analysis

## Abstract

Nuclear magnetic resonance (NMR) spectroscopy is a powerful tool for process monitoring. However, the quantitative analysis of highly diluted components or of components in small sample volumes is difficult with this analytical method due to its inherent lack of sensitivity. The hyperpolarization technique Overhauser dynamic nuclear polarization (ODNP) holds promise to solve this problem because it can provide strong signal enhancements. Furthermore, ODNP operates on short time scales and can therefore be used on flowing samples. Despite these advantages, to our knowledge, ODNP has never been applied for quantitative analysis of mixtures–probably because NMR signal enhancements by ODNP can vary greatly for different molecules, making quantitative analysis of mixtures difficult. We demonstrated that this problem can be solved by a robust calibration: three binary mixtures were studied as test cases in a wide range of concentrations by ODNP‐enhanced 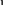
H and 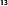
C NMR spectroscopy in continuous‐flow experiments with a benchtop NMR spectrometer using a new tailored calibration procedure. We show that quantitative analysis with ODNP‐enhanced NMR spectroscopy is possible even under these challenging conditions.

## Graphical abstract

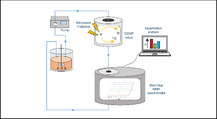

## 1 Introduction

Nuclear magnetic resonance (NMR) spectroscopy is a powerful tool that enables qualitative and quantitative analysis of complex mixtures.^[^ ^1^, ^2^, ^3^ ^]^ It is a noninvasive technique and widely used in science and engineering. Benchtop NMR spectrometers are particularly attractive because they are compact, robust and easy to maintain, so that they can be used in many environments.^[^ ^4^, ^5^, ^6^ ^]^ However, sensitivity is an issue in NMR spectroscopy and particularly relevant in benchtop NMR spectroscopy due to the low magnetic field.

Hyperpolarization techniques,^[^ ^7^ ^]^ such as para‐hydrogen induced polarization (PHIP),^[^ ^8^, ^9^, ^10^, ^11^ ^]^ optical pumping^[^ ^12^ ^]^ and Overhauser dynamic nuclear polarization (ODNP)^[^ ^13^ ^]^ are promising approaches to enhance signals up to a thousandfold and can be a game changer for sensitivity in NMR spectroscopy. Among these, ODNP is particularly attractive because of its ability to hyperpolarize a wide range of molecules and because it is fast, so that it can be used in continuous‐flow setups for real‐time process monitoring.^[^ ^14^, ^15^, ^16^, ^17^, ^18^, ^19^, ^20^, ^21^, ^22^ ^]^ In ODNP, the high polarization of electron spins is transferred to nuclear spins by the application of microwave irradiation, allowing theoretical signal enhancements of 658 on the 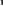
H nucleus and 2640 on the 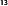
C nucleus.^[^ ^23^, ^24^, ^25^, ^26^, ^27^ ^]^ Stable radicals, such as nitroxides, are added to the sample as a source of electron spin polarization.^[^ ^28^, ^29^, ^30^ ^]^ For an application in process monitoring, it is advantageous to immobilize the radicals in a fixed bed, which allows flow‐induced separation of the radicals and thus a measurement of a radical free sample.^[^ ^14^, ^15^, ^17^, ^18^ ^]^ This avoids chemical contamination of the sample and interference of the radicals with NMR detection.

However, the quantitative analysis of hyperpolarized mixtures is challenging because the integral of the signal does not only depend on the number of spins in the sample but also on the polarization level. In ODNP, unequal nuclear spin polarization levels are observed for different components of mixtures and sometimes even for different nuclei on the same molecule, due to different hyperfine interactions of the molecular sites with the radicals. This prevents applying the common quantification based only on the peak integrals. Different methods to solve the problem of unequal polarization levels have been described in the literature: the signal amplification by reversible exchange (SABRE) hyperpolarization technique uses a second ligand, which binds at the mediating metal complex to achieve a linear dependence of the SABRE signal on the concentration of the studied component.^[^ ^9^, ^31^ ^]^ Using a calibration, the SABRE technique has been applied successfully, e.g. to quantify metabolites in urine samples or sugars solved in dimethylformamide.^[^ ^32^, ^33^ ^]^ A different approach has been developed for dissolution DNP (dDNP) and applied for the quantification of metabolic pathways and networks using an internal standard.^[^ ^34^, ^35^, ^36^, ^37^, ^38^, ^39^, ^40^, ^41^, ^42^, ^43^, ^44^ ^]^ Although originally proposed for biomedical applications, including in vivo imaging, applications of dDNP for online monitoring of chemical and biological reactions have emerged.^[^ ^45^, ^46^ ^]^ However, both SABRE and dDNP have drawbacks in online applications: SABRE can only be applied to molecules that are able to bind to the metal complex required for polarization transfer or that enable proton exchange, while dDNP is limited to the detection of very fast processes and cannot be used in continuous‐flow applications because the spin polarization produced is non‐renewable, allowing only studies on the time scale of the $T_{1}$ time of the observed nucleus. Hyperpolarization by ODNP is particularly suitable in this respect because it can polarize a wide range of different molecules at different concentrations, works also in continuous‐flow, and is technically straightforward. In a recent work by van der Ham,^[^ ^47^ ^]^ an approach was demonstrated which uses an interrupted ODNP hyperpolarization scheme to quantify ODNP‐enhanced 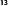
C NMR spectra of pure substances measured at high magnetic field (i.e. 9.4 T), and at rest. However, this approach requires the acquisition of several ODNP‐enhanced spectra with increasing MW irradiation times to allow for back‐extrapolation to zero irradiation times to extract quantitative information. Due to the need for multiple acquisitions, this approach might not be suitable for process and reaction monitoring.

In previous works from our group, we have systematically investigated various parameters affecting the performance of both 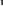
H and 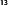
C ODNP in continuous‐flow experiments.^[^ ^17^, ^18^, ^48^ ^]^ In the present work, we report on the quantitative analysis of ODNP hyperpolarized mixtures with single‐scan 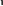
H and 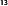
C NMR spectroscopy. To the best of our knowledge, this is the first study that reports on this new option for quantitative hyperpolarized NMR for the concentration determination of mixtures. To demonstrate the feasibility of this approach, we have carried out 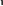
H and 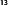
C ODNP experiments on three binary mixtures over a wide concentration range without the use of any internal standard. The experiments were performed in continuous‐flow. Immobilized radical matrices were used for the ODNP build‐up and the hyperpolarized mixtures were analyzed with a benchtop NMR spectrometer. It is shown that no tedious study of the different factors that influence ODNP (coupling, leakage and saturation factor) is necessary for the quantification. The effect of uneven hyperpolarization on the different molecules was addressed by a calibration that was tailored for the application to ODNP experiments and works reliably in the entire concentration range.

## 2 Experimental Section

### 2.1 Chemicals and Materials

Three binary systems were investigated (the suppliers and the purities of the chemicals are given in the Supporting Information): a) System 1: acetonitrile + water (ACN + W); b) System 2: acetonitrile + 1,4‐dioxane (ACN + DX); c) System 3: acetonitrile + chloroform (ACN + CF).

For each system, mixtures of different composition were prepared gravimetrically using a laboratory balance (Delta Range XS603S, Mettler Toledo, accuracy: ±0.001 g). The uncertainty of the concentrations obtained gravimetrically is ±0.001 mol mol^−1^. The immobilized paramagnetic radical matrix used in this study is the same as in our previous works and consists of the nitroxide radical TEMPO immobilized via a polymeric linker on controlled porous glass beads.^[^ ^18^, ^48^ ^]^ More details on the radical matrix are given in the Supporting Information.

### 2.2 Experimental Setup and Procedure

**Figure 1:**
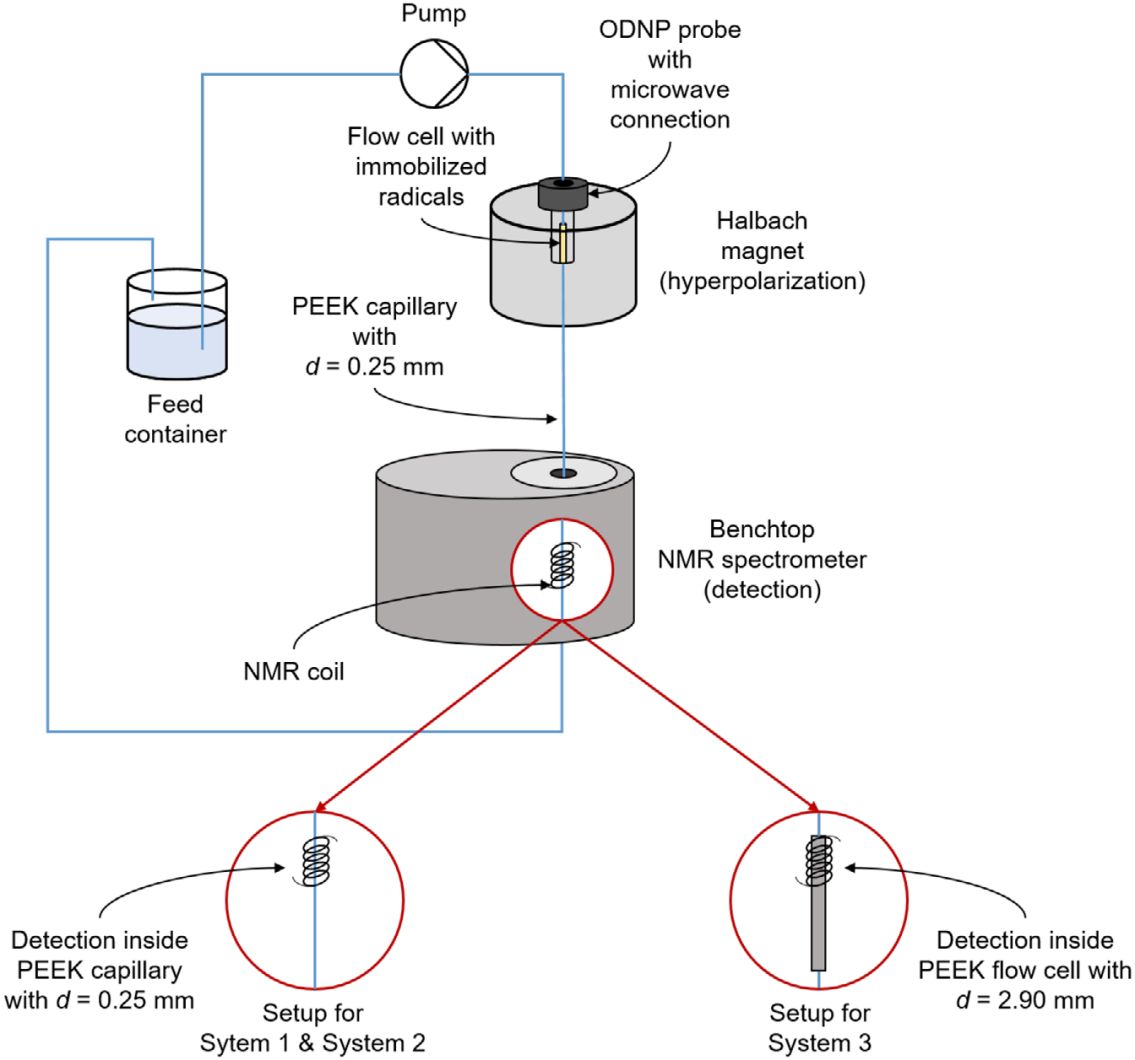
Scheme of the experimental setup for the continuous‐flow ODNP experiments.

**Figure** **1** illustrates the setup for the continuous‐flow ODNP experiments. The setup is adapted from previous work of our group.^[^ ^17^, ^48^ ^]^ It is only briefly described here, details as well as component specifications are given in the Supporting Information.

The main components of the setup are a custom‐built Halbach magnet (magnetic field strength 0.35 T) and a Magritek benchtop NMR spectrometer (Spinsolve Carbon, magnetic field strength 1.0 T). The sample mixture was pumped from a storage vessel to the ODNP probe inside the Halbach magnet containing the radical matrix in a fixed bed. Inside the ODNP probe, the sample was irradiated with microwave (MW) radiation at a frequency of 9.687 GHz to accomplish the hyperpolarization. After hyperpolarization, the sample flows through a PEEK capillary to the benchtop NMR spectrometer which was placed directly under the Halbach magnet. The inner diameter of the capillary connecting the ODNP probe and the detection cell of the benchtop NMR spectrometer was 0.25 mm, its length 0.52 m.


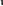
H NMR experiments with ODNP hyperpolarization (referred to as 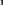
H NMR ODNP) were carried out for all systems, 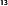
C NMR experiments with ODNP hyperpolarization (referred to as 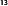
C NMR ODNP) were additionally carried out for System 3. Furthermore, corresponding thermally polarized 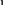
H and 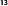
C NMR experiments without MW irradiation (referred to as 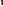
H NMR and 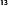
C NMR) were carried out to enable the calculation of the signal enhancement. Hence, there are four data sets: Three for the 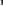
H NMR ODNP experiments in the Systems 1 to 3 and another one for the 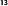
C NMR ODNP experiments in the System 3. All ODNP experiments were conducted with a single scan in continuous‐flow. Details of the experimental procedure as well as the calculation of the signal enhancement are provided in the Supporting Information.

Two different detection cells were used: For the studies of System 1 and System 2, a PEEK‐capillary with an inner diameter of 0.25 mm was used, since only 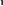
H NMR experiments, which yield large signals, were performed;^[^ ^17^ ^]^ for System 3, a custom‐built PEEK‐detection cell with an inner diameter of 2.90 mm was used to enable analysis by 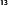
C NMR spectroscopy without the use of isotopically enriched samples.^[^ ^48^ ^]^ After passing the NMR spectrometer the sample was recycled to the storage vessel.

The flow rate $\dot{V}$ and the MW power *P* were set individually for each system based on experience from previous works.^[^ ^17^, ^48^ ^]^ The experimental parameters are summarized in **Table** **1**, which also provides the corresponding flow velocities *v* (calculated assuming plug‐flow) in the capillary between the ODNP probe and the NMR detection cell. The MW irradiation was activated 2 s before signal acquisition to allow sufficient ODNP hyperpolarization build‐up. The detection cell used for system 3 had an inner diameter of 2.9 mm, which was necessary to enable the natural abundance 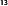
C NMR experiments described later. As a result, the 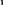
H NMR experiments for this system were conducted at a significantly higher flow velocity compared to System 1 and System 2, in order to ensure that the larger detection cell was filled within the 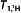 relaxation time of the molecules. However, this might result in an incomplete hyperpolarization build‐up for system 3.

**Table 1:** Experimental parameters: flow rate $\dot{V}$ and microwave power *P* for each studied system. Also the flow velocity *v* in the capillary between the ODNP probe and the NMR detection cell is provided. *d* is the inner diameter of the detection cell.

System	NMR	$\dot{V}$/ml min 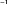	*v*/m s 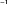	*P*/W	*d*/mm
1, 2	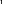 H	1.0	0.34	10	0.25
3	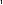 H	7.0	2.38	5	2.90
	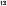 C	1.0	0.34	5	2.90

The (uncorrected) mole fraction $x_{i}^{\text{ODNP}}$ of component *i* in the binary systems was determined from the normalized peak integral with respect to the second component *j* in that system (see Equation (1)).

(1) $$x_{i}^{\text{ODNP}}=\frac{\frac{I_{i}^{\text{ODNP}}}{N_{i}}}{\frac{I_{i}^{\text{ODNP}}}{N_{i}}+\frac{I_{j}^{\text{ODNP}}}{N_{j}}}$$

where $I_{i}^{\text{ODNP}}$ denotes the integral of the signal of the component *i* and $N_{i}$ is the number of protons or carbon nuclei assigned to this signal. All 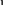
H NMR ODNP experiments were repeated 5 times, while 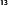
C NMR ODNP experiments were repeated 3 times. From the results the mean value $x_{i}^{\text{ODNP}}$ and the standard deviation, that are reported here, were calculated.

### 2.3 Calibration Function

**Figure 2:**
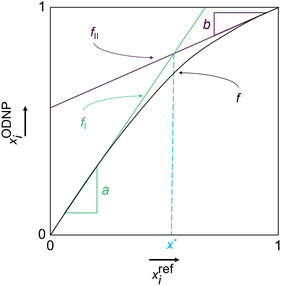
Illustration of the calibration function that relates $x_{i}^{\text{ODNP}}$ and $x_{i}^{\text{ref}}$. The calibration function *f* is basically a combination of two linear functions (green and purple) with a smooth transition between both.

Unequal hyperpolarization efficiencies for different components as well as differences in the hyperpolarization losses due to relaxation processes during the transport to the NMR detection result in differences between $x_{i}^{\text{ODNP}}$ and the true concentration. In this work, we take the gravimetrical concentration $x_{i}^{\text{ref}}$ as ground truth. The concentrations $x_{i}^{\text{ODNP}}$ obtained from the 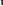
H NMR ODNP and 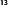
C NMR ODNP experiments were correlated to $x_{i}^{\text{ref}}$ of the corresponding mixture. A typical result for the relation of $x_{i}^{\text{ref}}$ and $x_{i}^{\text{ODNP}}$ is given in **Figure** **2**.

In both corners representing pure compounds, the relation between $x_{i}^{\text{ref}}$ and $x_{i}^{\text{ODNP}}$ is basically linear, but the slopes of the linear functions differ. At intermediate concentrations, there is a smooth transition between these two lines. This observation has led us to propose the empirical correlation function which is given in Equation (2). The function was chosen after preliminary tests with the aim of representing the observed relations $x_{i}^{\text{ODNP}}(x_{i}^{\text{ref}})$ with a flexible function with a minimal number of parameters.

(2) $$x_{i}^{\text{ODNP}}=f(x_{i}^{\text{ref}})=w_{I}(x_{i}^{\text{ref}})\cdot f_{I}(x_{i}^{\text{ref}})+w_{\text{II}}(x_{i}^{\text{ref}})\cdot f_{\text{II}}(x_{i}^{\text{ref}})$$

with:

(3) $$f_{I}(x_{i}^{\text{ref}})=a\cdot x_{i}^{\text{ref}}$$

(4) $$f_{\text{II}}(x_{i}^{\text{ref}})=(1-b)+b\cdot x_{i}^{\text{ref}}$$

The parameters *a* and *b* describe the slopes of the linear calibration curves in the highly diluted regions and are fitted to the experimental data points. The weights $w_{I}$ and $w_{\text{II}}$ are defined such that the following conditions are satisfied:

$$w_{I}(0)=1\text{ and }w_{I}(1)=0$$

$$w_{\text{II}}(0)=0\text{ and }w_{\text{II}}(1)=1$$

The weight functions are given in Equation (5) and Equation (6).

(5) $$w_{I}(x_{i}^{\text{ref}})=\frac{\tanh\left(c\left(x_{i}^{*}-x_{i}^{\text{ref}}\right)\right)-\tanh\left(c\left(x_{i}^{*}-1\right)\right)}{\tanh\left(c\left(x_{i}^{*}-0\right)\right)-\tanh\left(c\left(x_{i}^{*}-1\right)\right)}$$

(6) $$w_{\text{II}}(x_{i}^{\text{ref}})=\frac{\tanh\left(c\left(x_{i}^{\text{ref}}-x_{i}^{*}\right)\right)-\tanh\left(c\left(0-x_{i}^{*}\right)\right)}{\tanh\left(c\left(1-x_{i}^{*}\right)\right)-\tanh\left(c\left(0-x_{i}^{*}\right)\right)}$$

where $x_{i}^{*}$ is

(7) $$x_{i}^{*}=\frac{1-b}{a-b}$$

which is the value of $x_{i}^{\text{ref}}$ for which the two functions $f_{I}(x_{i}^{\text{ref}})$ and $f_{\text{II}}(x_{i}^{\text{ref}})$ intersect. The parameter *c* controls the steepness of the transition between these two branches of the calibration curve. It can be adjusted, but also a default value can be chosen, as it was done in the present work. Based on preliminary studies the value c=3 was selected and used for all evaluations. Hence, even though the mathematical form of the calibration curve described by Equation ((2), (3), (4), (5), (6), (7)) may seem intricate, the function itself is simple to handle and has only two adjustable parameters *a* and *b* (or three if *c* is not set). Furthermore, it also contains the fully linear correlation as a limiting case (for a=b). Formally, the component *i* can be chosen to be either 1 or 2. As the mole fractions of the two components of the binary mixture sum up to 1, both choices are equivalent. In the present work, we always refer to component 1, which was ACN in all three studied systems.

For each of the studied systems, the parameters *a* and *b* were determined from a fit to the experimental data points, which was performed with the nonlinear least‐squares solver *lsqcurvefit* of MATLAB using the following target function

(8) $$S=\sum_{k=1}^{M}{\left(f\left(x_{\text{ACN},k}^{\text{ref}}\right)-x_{\text{ACN},k}^{\text{ODNP}}\right)}^{2}$$

where *M* is the number of data points in the system.

To quantify the accuracy of the obtained correlation, the mean absolute error (MAE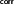) is calculated according to Equation (9).

(9) $${\text{MAE}}_{\text{corr}}=\frac{\sum_{k=1}^{M}\left|f\left(x_{\text{ACN},k}^{\text{ref}}\right)-x_{\text{ACN},k}^{\text{ODNP}}\right|}{M}$$

The robustness of this calibration approach was evaluated using a leave‐one‐out (LOO) analysis^[^ ^49^ ^]^: One data point (here: the result for one mixture $k^{*}$) of the data set (here: the set of results for a given system) was left out, while the others were used for the fit. The obtained calibration function was then used to calculate $\left|f\left(x_{\text{ACN},k^{*}}^{\text{ref}}\right)-x_{\text{ACN},k^{*}}^{\text{ODNP}}\right|$. This procedure was repeated for all *M* data points (mixtures) of the studied system and the mean absolute error of the LOO analysis (MAE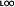) was calculated in the same way as for MAE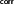, cf. Equation (9).

## 3 Results and Discussion

The signal integrals from the ODNP experiments were used to calculate $x_{\text{ACN}}^{\text{ODNP}}$ for all data points using Equation (1). Furthermore, for all data points $x_{\text{ACN}}^{\text{ref}}$ is known. Based on these data, for the studied system the calibration function Equation ((2), (3), (4), (5), (6), (7)) was fitted to the data points ($x_{\text{ACN}}^{\text{ref}}$, $x_{\text{ACN}}^{\text{ODNP}}$) and the LOO analysis was carried out, yielding MAE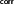 and MAE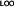. In **Table** **2**, we report these values together with the numbers for *a* and *b* found from the correlation of the full data set. The values for MAE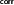 are below about 0.02 mol mol^−1^ for the 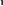
H NMR as well as for the 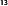
C NMR data, which is remarkable, considering the fact that the data stem from single‐scan experiments in flow. The values of MAE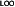 are somewhat higher, but underline the robustness of the method. A detailed discussion of the results is given in the next sections. Additional data on the signal integrals, the signal enhancements, and the spin‐lattice relaxation time $T_{1}$ as well as the relative deviations of data points from the calibration curve are reported in the Supporting Information.

**Table 2:** Results from the fits of the calibration curves. Parameters *a* and *b* of the calibration function (equation (2) ‐ (7)) were obtained from a fit to all available experimental data points of the studied system. Additionally, the resulting value of $x_{\text{ACN}}^{*}$ is provided. The number for *c* was not adjusted to the data and set to c=3 for all systems. The numbers for the mean absolute error of the correlation of all data points (MAE) and that found in the leave‐one‐out analysis (MAE) are also reported.

System	NMR	*a*	*b*	$x_{\text{ACN}}^{*}$/	MAE 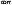 /	MAE 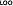 /
				mol mol 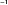	mol mol 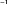	mol mol 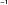
System 1	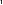 H	1.3023	0.4959	0.625	0.007	0.013
System 2	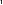 H	1.0498	0.9467	0.517	0.018	0.015
System 3	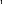 H	0.6624	1.1946	0.366	0.021	0.039
	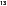 C	0.5064	2.1848	0.706	0.023	0.040

In general, there are two effects that affect the ODNP enhancement and consequently may result in a nonlinear calibration curve: 1) the different types and strengths of hyperfine interactions of the molecules with the radical; 2) the unequal hyperpolarization losses due to differences in $T_{1}$ during the transport from the fixed bed to the benchtop NMR spectrometer. From a physical point of view, the two linear segments of the calibration function may be interpreted as indicative of concentration ranges in which one component primarily interacts with the radicals. In ODNP terminology,^[^ ^25^ ^]^ this is primarily reflected by the coupling factor, which describes the hyperfine interaction between the radical and the molecule of interest. The coupling factor is mainly influenced by the accessibility of the radical site and the molecular dynamics of the analyte–radical system. Changes in bulk viscosity (and consequently in the relevant molecular dynamics and correlation times) play a major role in determining the coupling factor and thus the ODNP enhancement.

### 3.1 System 1: Acetonitrile (ACN) + Water (W)

**Figure 3:**
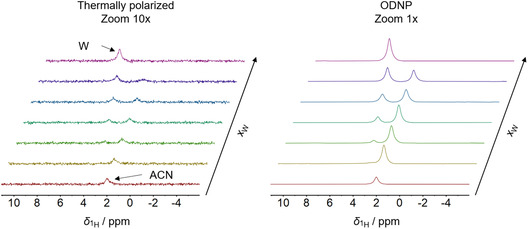
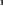
H NMR spectra of System 1: ACN + W for mixtures with different composition acquired with a single scan in continuous‐flow (flow velocity *v* = 0.34 m s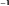).

**Figure** **3** shows the 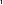
H NMR spectra of the different studied mixtures of System 1: ACN + W obtained with the 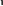
H NMR ODNP experiments in continuous‐flow. For comparison, also the results of the corresponding thermally polarized experiments are shown. Two singlet peaks can be identified which are assigned to ACN and W. Both peaks are broad due to flow effects that reduce the spin‐spin relaxation time $T_{2}$.^[^ ^4^ ^]^ However, the peaks do not overlap, allowing a quantification by direct integration of the signals. The thermally polarized 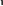
H NMR experiments show a low signal‐to‐noise ratio (SNR). By switching on the MW and performing the 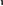
H NMR ODNP experiment, a significant improvement in the SNR is achieved. The average signal enhancements were 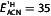 for ACN and 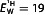 for W for System 1.

In both types of experiments, the signal integral of W increases linearly as more W is added to the mixture. However, for ACN a deviation from the linear relationship is observed in the 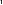
H NMR ODNP experiment: the maximum signal integral for ACN is not found for the pure substance, but for a mole fraction of $x_{\text{ACN}}^{\text{ref}}=0.584$ mol mol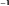. This is due to a change in the molecular dynamics of the system as the composition of the samples changes, which affects the interaction of the molecules with the radicals and, hence, the corresponding signal enhancement.

**Figure 4:**
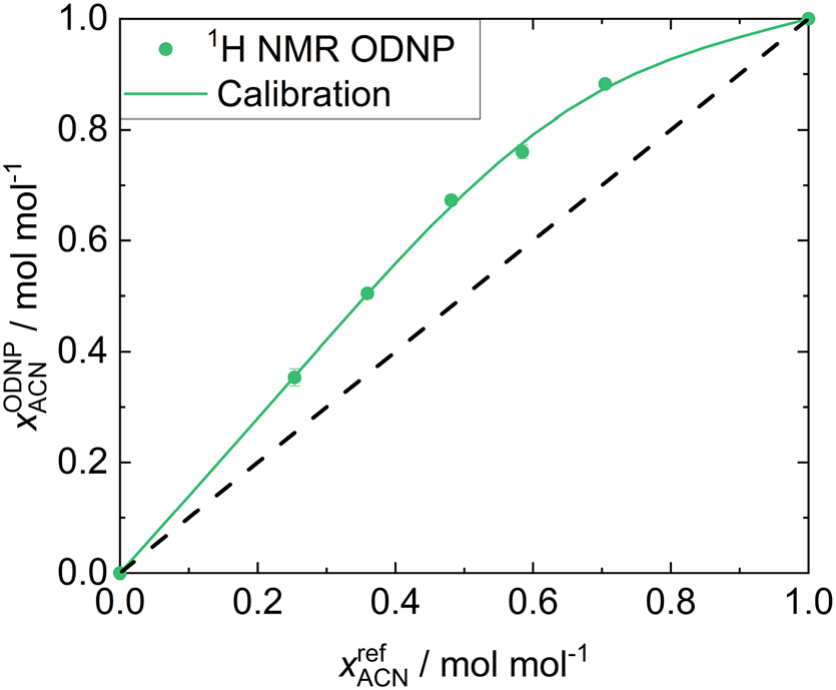
Results for System 1: ACN + W. $x_{\text{ACN}}^{\text{ODNP}}$ obtained in the 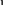
H NMR ODNP experiments (symbols) are plotted over the gravimetric reference values $x_{\text{ACN}}^{\text{ref}}$. The calibration curve (solid line) resulting from the fit to all data as well as the ideal diagonal line (dashed line) are given.

**Figure** **4** shows the results of the quantitative analysis of System 1 with the 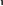
H NMR ODNP experiments. The results for $x_{\text{ACN}}^{\text{ODNP}}$ obtained in the 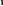
H NMR ODNP experiment are plotted over the gravimetric reference value $x_{\text{ACN}}^{\text{ref}}$ and the calibration curve resulting from the fit to all data points is shown.

The concentrations of ACN were systematically overestimated in the ODNP experiment (and, as consequence, those for the concentrations of W were systematically underestimated). This is a result of the significant difference in the observed signal enhancements which are mainly due to differences in the 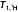 values between ACN and W. The $T_{1}$ values of ACN are much larger than those of W, see Supporting Information. This leads to lower hyperpolarization losses during the transport from the fixed bed to the detection zone for ACN compared to those for W.

However, the excellent description of the results by the correlation is evident in Figure 4. The calibration function fits the experimental data points very well. In particular, the almost linear data for ACN mole fractions below about 0.5 mol mol^−1^ is described very well. The MAE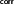 is less than 0.007 mol mol^−1^, which is a very good result for quantitative NMR measured in a 0.25 mm capillary in a benchtop NMR spectrometer in continuous‐flow. The MAE obtained in the LOO analysis is only 0.013 mol mol^−1^, indicating that our approach provides a robust calibration function.

### 3.2 System 2: Acetonitrile (ACN) + 1,4‐Dioxane (DX)

**Figure 5:**
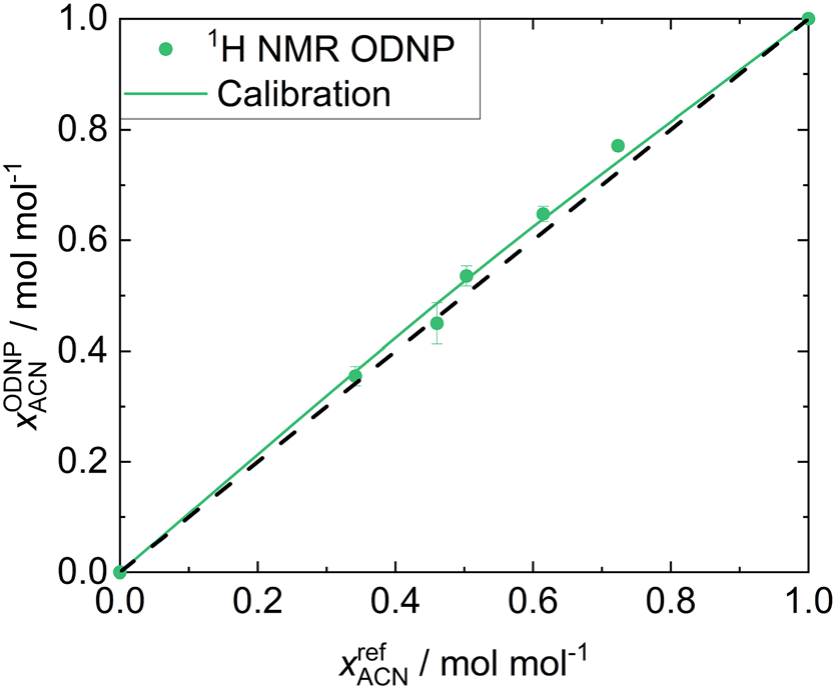
Results for System 2: ACN + DX. $x_{\text{ACN}}^{\text{ODNP}}$ obtained in the 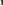
H NMR ODNP experiments (symbols) are plotted over the gravimetric reference values $x_{\text{ACN}}^{\text{ref}}$. The calibration curve (solid line) resulting from the fit to all data as well as the ideal diagonal line (dashed line) are given.

**Figure** **5** shows the results of the quantitative analysis with the 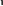
H NMR ODNP experiment and the calibration curve for System 2: ACN + DX. The 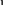
H NMR spectra of the thermally polarized 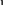
H NMR and 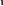
H NMR ODNP experiments in continuous‐flow are provided in the Supporting Information.

In Figure 5, a linear dependence of $x_{\text{ACN}}^{\text{ODNP}}$ on $x_{\text{ACN}}^{\text{ref}}$ can be observed over almost the entire concentration range. In contrast to System 1, the results are close to the ideal diagonal line. Also this behavior is described very well by the calibration function. The MAE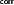 is 0.018 mol mol^−1^ and the MAE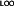 is 0.015 mol mol^−1^, respectively, which is a good result for quantitative benchtop NMR of fast flowing samples.

The slopes of both linear functions and, thus, the parameters *a* and *b* do not differ much from each other for this system. The calibration function collapses to a almost linear correlation. There are two reasons for this observation: First, the difference in the $T_{1}$ values of ACN and DX is small compared to System 1 (see Supporting Information). Therefore, hyperpolarization losses during the transport are not relevant for the quantification in this system. Second, the hyperfine interactions of both molecules with the radicals are expected to be similar. This is supported by the similar enhancement values obtained for ACN and DX in the different mixtures (see Supporting Information).

### 3.3 System 3: Acetonitrile (ACN) + Chloroform (CF)

**Figure 6:**
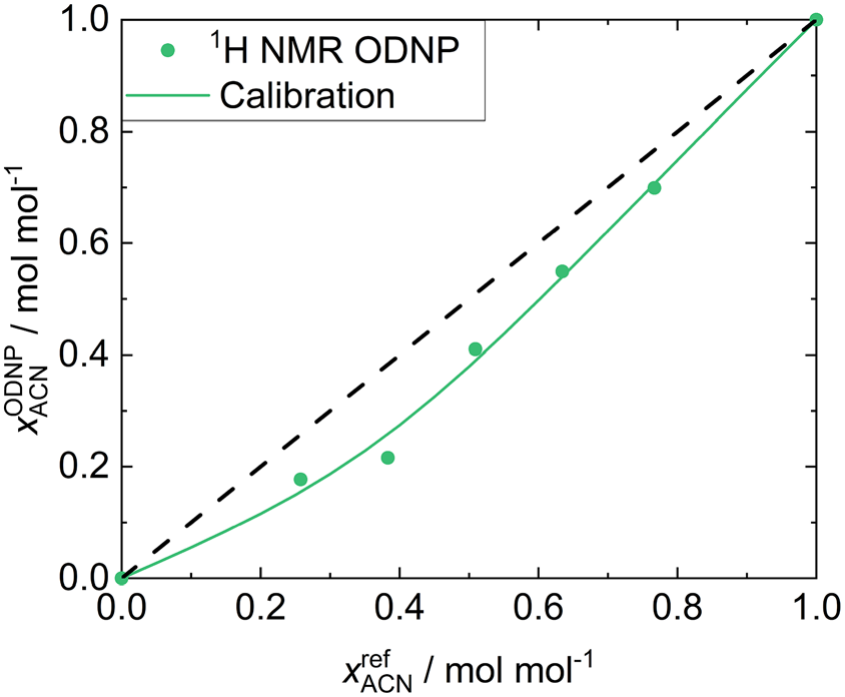
Results for System 3: ACN + CF. $x_{\text{ACN}}^{\text{ODNP}}$ obtained in the 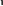
H NMR ODNP experiments (symbols) are plotted over the gravimetric reference values $x_{\text{ACN}}^{\text{ref}}$. The calibration curve (solid line) resulting from the fit to all data as well as the ideal diagonal line (dashed line) are given.

**Figure** **6** shows the results of the quantitative analysis with the 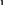
H NMR ODNP experiments and the calibration curve for System 3: ACN + CF. Again, the 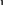
H NMR spectra of the thermally polarized 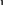
H NMR and 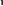
H NMR ODNP experiments in continuous‐flow are provided in the Supporting Information. The detection cell for this system had an inner diameter of 2.9 mm to enable the 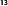
C NMR experiments at natural abundance described later. Therefore, the 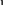
H NMR experiments for this system were carried out at a much higher flow velocity than for System 1 and System 2 (v=0.34 m s^−1^ for System 1 and System 2 compared to v=2.38 m s^−1^ for System 3) to allow the larger detection cell to be filled within the $T_{{\text{1,}}^{1}H}$ relaxation time of the molecules. Furthermore, the MW power was reduced in these experiments because of the high volatility of CF, resulting in lower enhancement values and lower SNR. Moreover, the sudden expansion at the inlet of the detection cell from 0.25 to 2.9 mm leads to the formation of a jet‐flow.^[^ ^48^ ^]^ Therefore, a high degree of back‐mixing is present in the detection cell, which further reduces hyperpolarization and affects quantification.

Despite these challenges, the calibration function correlates the data very well. The MAE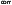 is 0.021 mol mol^−1^. However, an outlier is identified at an ACN mole fraction of $x_{\text{ACN}}^{\text{ref}}=0.383$ mol mol^−1^. As a consequence, a larger MAE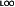 of 0.039 mol mol^−1^ is found. Nevertheless, the results underline the robustness of our calibration method.

In contrast to System 1, the ACN concentration is systematically underestimated by the 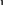
H NMR ODNP experiment whereas the CF concentration is overestimated. This observation can be explained by the $T_{1}$ values, which are reversed in this system: ACN has a significantly shorter 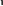
H spin‐lattice relaxation time than CF, resulting in larger hyperpolarization losses for ACN.

**Figure 7:**
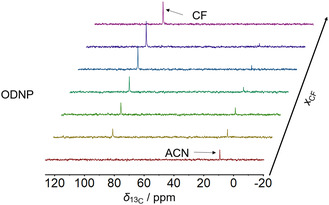
ODNP‐enhanced 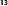
C NMR spectra of System 3: ACN + CF for mixtures with different composition acquired with a single scan in continuous‐flow (flow velocity v=0.34 m s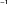).

**Figure** **7** shows 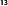
C NMR spectra of the different mixtures of System 3 obtained by the 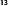
C NMR ODNP experiment with only one scan in continuous‐flow. Without ODNP a similar experiment would have yielded no signals at all. The flow rate for the 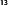
C NMR experiments of this system was reduced (v=0.34 m s^−1^) compared to the one for the 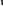
H NMR experiments due to the much longer $T_{{\text{1,}}^{13}C}$ relaxation times. The two singlet peaks in the spectra can be clearly assigned to the methyl group of ACN and CF, respectively. The large chemical shift dispersion prevents peak overlap, which is particularly beneficial for benchtop NMR spectroscopy. A mean signal enhancement of 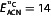 and 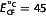 was achieved. The higher enhancement of CF is a consequence of its molecular structure (electron withdrawing effect of the chlorine atoms), which results in a stronger hyperfine interaction to TEMPO radicals.^[^ ^15^, ^21^, ^22^, ^50^ ^]^

**Figure 8:**
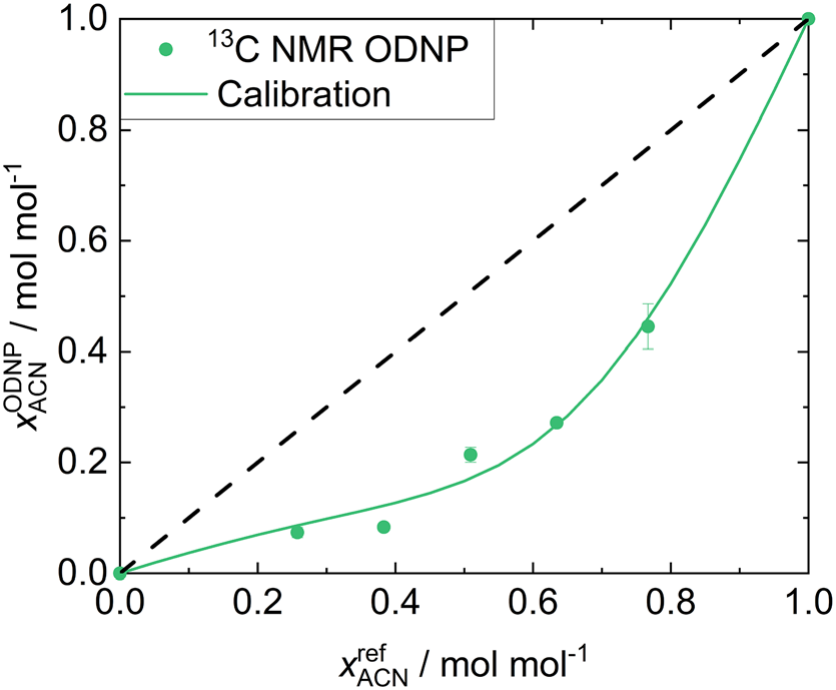
Results for System 3: ACN + CF. $x_{\text{ACN}}^{\text{ODNP}}$ obtained in the 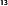
C NMR ODNP experiments (symbols) are plotted over the gravimetric reference values $x_{\text{ACN}}^{\text{ref}}$. The calibration curve (solid line) resulting from the fit to all data as well as the ideal diagonal line (dashed line) are given.

In **Figure** **8**, the results of the quantitative analysis with the 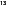
C NMR ODNP experiment and the calibration curve for System 3: ACN + CF are shown. Similar to the experiments with 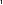
H NMR ODNP, an overestimation of CF is observed, which is explained by the larger ODNP enhancement of CF compared to ACN. Relaxation effects on the 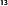
C nuclei must also be considered, as the chosen flow velocity of 0.34 m s^−1^ results in a mean transport time of 1.5 s. However, these effects are not as strong compared to the 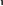
H NMR ODNP experiments, as the 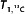 values are larger. An additional time delay occurs due to the filling of the large detection volume with hyperpolarized fluid. For the pure components ACN and CF the 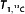 values were determined experimentally (for ACN: 15.4 s; for CF: 21.7 s^[^ ^48^ ^]^), which leads to higher polarization losses for ACN. The combination of both effects (stronger hyperfine interaction and larger $T_{1}$ time) leads to an overestimation of the CF concentration, and consequently an underestimation of the ACN concentration, as observed in Figure 8 and as expected.

For the 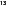
C NMR ODNP experiments, the obtained $x_{\text{ACN}}^{\text{ODNP}}$ mole fractions deviate strongly from the diagonal line. The calibration function is still able to correlate the experimental data satisfactorily, but the MAE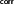 of 0.023 mol mol^−1^ and the MAE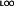 of 0.040 mol mol^−1^ are the highest of all quantitative analyses. This is explained by the significantly lower SNRs of the 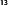
C NMR spectra compared to those of the 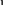
H NMR spectra, which is due to the low natural abundance of 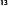
C, resulting in larger uncertainties. In addition, the quantification is disturbed by the jet‐flow present in the detection cell. Improving the accuracy of 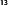
C NMR quantification with ODNP by improving the experimental setting is the subject of ongoing research in our laboratory. However, even the first experiments demonstrate that ODNP enables a reasonable quantitative analysis of mixtures by single‐scan benchtop 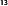
C NMR spectroscopy in continuous‐flow, which is impossible without ODNP. This is particularly useful when NMR signals overlap in the 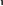
H NMR spectrum as it is often the case for mixtures measured with benchtop NMR spectrometers.

## 4 Conclusions

ODNP can be used for overcoming sensitivity issues of NMR spectroscopy as it greatly increases the signal intensity and thereby the SNR. This is especially important for reaction monitoring applications, where the analysis must often be carried out with single‐scan experiments. However, as the signal enhancement is generally different for different components and many factors influence the individual enhancements, a quantification of NMR spectra obtained with ODNP is difficult. To the best of our knowledge, this is the first publication in which this issue is tackled for the quantification of mixtures. We have shown that a quantification of both 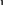
H NMR and 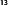
C NMR spectra of mixtures obtained in continuous‐flow experiments with ODNP in a benchtop NMR spectrometer is possible using a simple calibration—without having to elucidate and quantify all effects that lead to the different signal enhancements for the different components. A special calibration function was developed for that purpose that describes the nonlinear data for the different studied binary systems very well with only two parameters. Our results indicate that the most important parameters that influence the enhancements and, hence, the type of calibration curve that is obtained, are the strengths of the hyperfine interaction of the components of the mixture with the radicals in the fixed bed and the polarization losses during transport from the fixed bed to the NMR detection, that basically depend on the $T_{1}$ time of the components. To apply ODNP for quantitative analysis in a flow setup as we have used it, we recommend the following procedure: in a preliminary study, suitable experimental parameters, such as the microwave power and flow rate should be determined. Alternatively, they could be set based on experience. For these parameters, a calibration can be carried out. Then, routine analysis is possible, including single‐scan experiments with 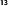
C NMR on benchtop instruments in continuous‐flow. In future work, a first application could be the monitoring of binary distillation processes, as these represent dynamic systems involving only two components, where the developed calibration function can be directly applied. Moreover, the presented approach should be extended and tested for multi‐component systems to further explore its versatility and applicability. A promising and straightforward application could be the monitoring of simple chemical reactions involving only a limited number of reactants. A particularly suitable test case could be an esterification reaction, where two reactants form two products, allowing for a well‐defined molecular environment. Additionally, diluted reactions with a large excess of solvent represent a compelling scenario. In such cases, the molecular dynamics should be largely independent of the reactant concentrations. Consequently, the ODNP signal would be expected to scale directly with the reactant concentration, offering a straightforward path toward quantification. These scenarios will be evaluated in future studies, building on the fact that the application of ODNP to quantitative flow NMR has now been successfully demonstrated.

## Conflict of Interest

The authors declare no conflict of interest.

## Supporting information

Supplementary Material

## Data Availability

The data that support the findings of this study are available from the corresponding author upon reasonable request.
