# Supplementary material for: Quantitative Nuclear Magnetic Resonance Spectroscopy with Overhauser Dynamic Nuclear Polarization
Source: Chemphyschem. 2025 Jul 18;26(16):e202401052. doi: 10.1002/cphc.202401052 (PMC12388179; doi:10.1002/cphc.202401052)
Supplement: Supplementary file 1 — Supplementary Material [file CPHC-26-e202401052-s001.pdf]

Quantitative NMR Spectroscopy with Overhauser Dynamic Nuclear Polarization - Supporting Information -

Johnnie Phuong, Raphael Kircher, Sarah Mross, Billy Salgado, Hans Hasse, and Kerstin Münnemann*

Laboratory of Engineering Thermodynamics (LTD), RPTU Kaiserslautern, Erwin-Schrödinger-Straße 44, 67663 Kaiserslautern, Germany

Laboratory of Advanced Spin Engineering - Magnetic Resonance (LASE-MR), RPTU Kaiserslautern, Gottlieb-Daimler-Straße 76, 67663 Kaiserslautern, Germany

E-mail: kerstin.muennemann@rptu.de

Experimental Section

Chemicals and Materials

Table SI.1 summarizes the chemicals which were used in this work without further purification. In addition, ultrapure water (W) was provided by Starpure's OmniaPure UV/UF-TOC water treatment system with a resistivity of 18.2 MΩ cm.

Table SI.1: Chemicals used in this work including the suppliers and the purities as specified by the suppliers.

Chemical	Supplier	Purity
Acetonitrile	Carl Roth	$\geq$ 99.9%
Chloroform	Merck	$\geq$ 99.0%
1,4-Dioxane	Sigma Aldrich	$\geq$ 99.8%

The immobilized radical matrix used in this work was made of nitroxide radicals glycidyloxy-tetramethylpiperidinyloxyl immobilized via a polyethylene-imine-linker (molecular mass 25,000 g mol-1) on aminopropyl-functionalized controlled porous glasses (CPG) with a pore size of 50 nm.

Experimental Setup

The liquid sample mixture was taken from a storage vessel (volume $V = 100$ ml) at ambient pressure and temperature. A double piston high pressure pump with damping piston (WA-Dose Plus HP, Flusys, accuracy: < 3 %), which was calibrated for a flow range of 0.5 to 10 ml min^{-1}, was used for the transport of the sample. The pressure increase by the pump was between 1 and 40 bar indicated by the pressure gauge integrated in the pump (accuracy: 0.5 %). The probe for ODNP hyperpolarization (Bruker BioSpin EN 4148X-MD4 electron nuclear double resonance ENDOR probe) was not thermostated; it was connected to the MW source (details of the hardware are given in Kircher et al.[1]). The paramagnetic fixed

bed was mounted in a PEEK tube (inner diameter 1.0 mm) and was installed inside the ODNP probe filling its 4 mm cavity resonator. For System 3, the 2.9 mm detection cell was positioned inside the benchtop NMR spectrometer so that the sensitive region of the NMR coil was located close to the expansion from 0.25 to 2.9 mm to minimize hyperpolarization losses by T_1 relaxation. A detailed description of the fluid characteristics of this setup is given in Phuong et al.[2]

Experimental Procedure and Calculation of the Signal Enhancements

All NMR experiments were controlled by the Spinsolve Expert software (Magritek). ^{1}H NMR ODNP experiments were performed with an acquisition time of 0.4 s, 2048 data points, 1 scan, and a 90° excitation pulse. ^{13}C NMR ODNP experiments were performed with an acquisition time of 1.6 s, 16 k data points, 1 scan and a 90° excitation pulse; an inverse-gated decoupling sequence (WALTZ-16) was applied during acquisition. To calculate the achieved ^{1}H ODNP signal enhancements ($E_i^{^1\mathrm{H}}$), ^{1}H NMR experiments with the same acquisition parameters were performed under the same flow conditions. For the ^{13}C ODNP signal enhancements ($E_i^{^{13}\mathrm{C}}$), the results of the ^{13}C NMR ODNP experiment were referenced to the ^{13}C NMR experiments which were acquired with the same acquisition parameters but with 256 scans in the absence of flow as the premagnetization is not sufficient for a detection of flowing samples. Manual correction of baseline, phase and peak integration was performed in MestReNova (Mestrelab Research).

For the calculation of the signal enhancement E of the ODNP experiments, the spectra were scaled to the same noise level. The signal and the noise of the thermally polarized NMR spectra as well as of the NMR spectra with ODNP enhancement were divided by the square root of the number of accumulated scans. The calculation of the signal enhancement is given in Equation (SI.1):

$$E = \frac{I^{\text{ODNP, scaled}}}{I^{\text{thermal, scaled}}} \cdot \frac{\sqrt{n^{\text{thermal}}}}{\sqrt{n^{\text{ODNP}}}} \tag{SI.1}$$

Here, $I^{\text{ODNP, scaled}}$ denotes the integral of the scaled signal obtained with ODNP enhancement, $I^{\text{thermal, scaled}}$ the integral of the scaled thermally polarized signal, and n the number of scans. Note that a correction for the receiver gain is not necessary since this parameter was kept constant in within the respective ^{1}H and ^{13}C experiments.

The error of the signal enhancements are calculated with an error propagation which is given in Equation (SI.2). For the estimation of ΔI^{ODNP} and $\Delta I^{\text{thermal}}$ the specific standard uncertainty of the experiments is applied.

$$\Delta E = \left| \left(\frac{\sqrt{n^{\text{thermal}}}}{\sqrt{n^{\text{ODNP}}}} \cdot \frac{1}{I^{\text{thermal, scaled}}} \right) \right| \cdot \Delta I^{\text{ODNP}} + \left| \left(- \frac{\sqrt{n^{\text{thermal}}}}{\sqrt{n^{\text{ODNP}}}} \cdot \frac{I^{\text{ODNP}}}{\left(I^{\text{thermal, scaled}}\right)^2} \right) \right| \cdot \Delta I^{\text{thermal}} \tag{SI.2}$$

Inversion recovery experiments were performed to determine the spin-lattice relaxation time $T_{1,\,^{1}\text{H}}$ of the ^{1}H nuclei for each mixture. The experiments were carried out with the standard operating software Spinsolve of the benchtop NMR spectrometer in NMR sample tubes with an inner diameter of 5 mm (Magritek) and were repeated 3 times.

Furthermore, the relative deviations of the data points from the calibration curve are calculated according to Equation (SI.3).

$$\varDelta_{\text{rel}} = \frac{f\left(x_{\text{ACN}}^{\text{ref}}\right) - x_{\text{ACN}}^{\text{ODNP}}}{x_{\text{ACN}}^{\text{ODNP}}} \cdot 100\% \tag{SI.3}$$

Results and Discussion

System 1: Acetonitrile (ACN) + Water (W)

In Figure SI.1, the obtained signal integrals of ACN and W of System 1, which are acquired with the ^{1}H NMR ODNP experiment, are shown as a function of the composition. The corresponding signal enhancements are displayed in Figure SI.2. The relative deviations of the data points from the calibration curve are shown in Figure SI.3. Table SI.2 provides the numerical values for the signal integrals and the uncorrected mole fractions x_i^{ODNP} acquired with the ^{1}H NMR ODNP experiment as well as the spin-lattice relaxation time $T_{1,\,^1\mathrm{H}}$ of ACN and W in the mixtures.

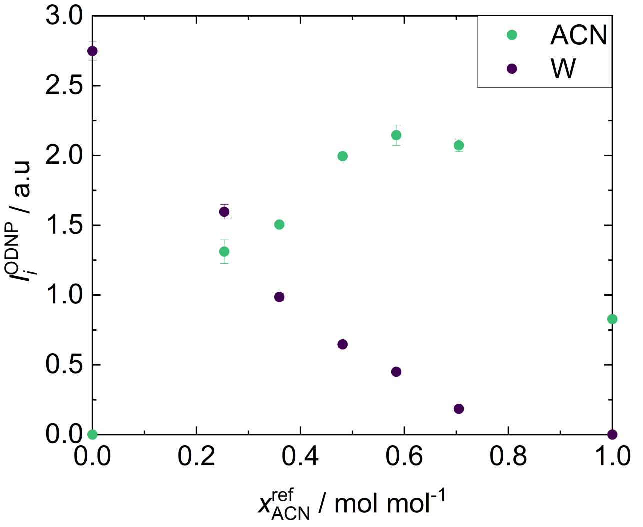

Figure SI.1: Signal integrals of ACN and W of System 1 as a function of the mixtures's composition acquired with the ^{1}H NMR ODNP experiment.

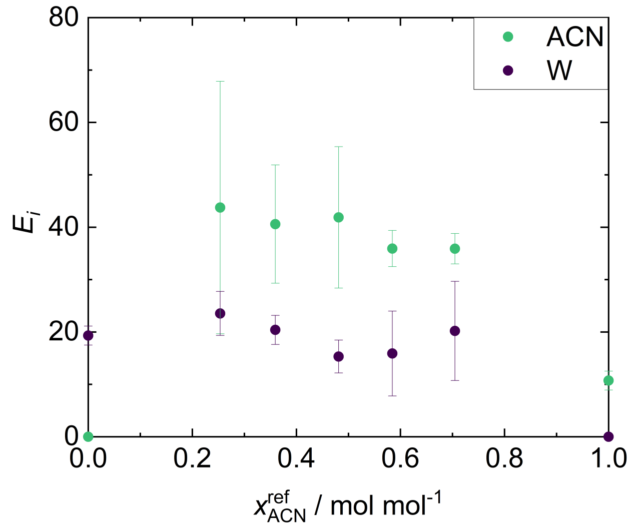

Figure SI.2: Signal enhancements of ACN and W of System 1 as a function of the mixtures's composition acquired with the ^{1}H NMR ODNP experiment.

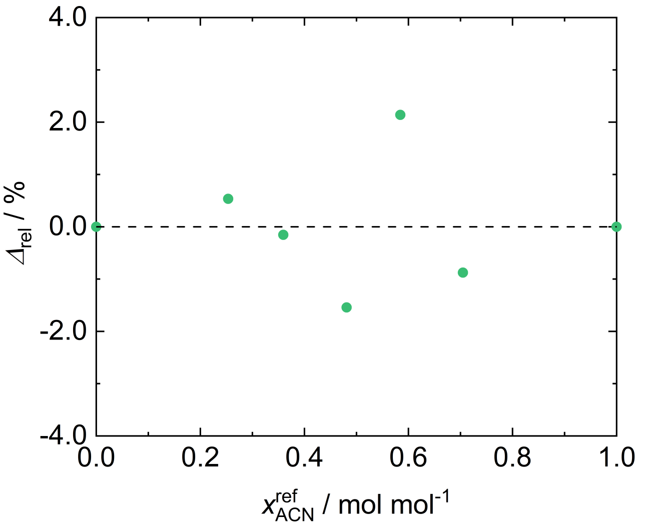

Figure SI.3: Relative deviations of the data points obtained with the ^{1}H NMR ODNP experiment from the calibration curve of System 1: ACN + W.

Table SI.2: Signal integrals I^{ODNP} of ACN and W of System 1 acquired with the ^{1}H NMR ODNP experiment, the signal enhancements $E^{^{1}\mathrm{H}}$ and the uncorrected mole fraction x_i^{ODNP} in dependence of the composition. Spin-lattice relaxation time $T_{1,\,^{1}\mathrm{H}}$ at a magnetic field strength of 1 T of selected mixtures are given. The ^{1}H NMR ODNP experiments were repeated 5 times; $T_{1,\,^{1}\mathrm{H}}$ were obtained with 3 repetitions. Standard uncertainties and errors are given, respectively.

Mixture	ACN				W		
x_{ACN} / mol mol^{-1}	I^{ODNP} / a.u.	$E^{^{1}\mathrm{H}}$	x^{ODNP} / mol mol^{-1}	$T_{1,\,^{1}\mathrm{H}}$ / s	I^{ODNP} / a.u.	$E^{^{1}\mathrm{H}}$	$T_{1,\,^{1}\mathrm{H}}$ / s
0	-	-	0	-	2.75±0.07	19.3±1.8	3.10±0.05
0.254	1.31±0.08	43.8±24.1	0.354±0.016	4.81±0.03	1.60±0.05	23.5±4.2	2.99±0.01
0.359	1.50±0.01	40.6±11.3	0.504±0.006	-	0.99±0.02	20.4±2.8	-
0.481	1.99±0.02	41.9±13.5	0.673±0.008	4.47±0.02	0.65±0.02	15.3±3.1	3.10±0.01
0.584	2.14±0.07	35.9±3.5	0.760±0.012	-	0.45±0.02	15.9±8.1	-
0.705	2.07±0.05	35.9±2.9	0.882±0.005	4.27±0.01	0.18±0.01	20.2±9.5	3.33±0.02
1	0.83±0.02	10.7±1.8	1	3.99±0.01	-	-	-

System 2: Acetonitrile (ACN) + 1,4-Dioxane (DX)

Figure SI.4 displays the ^{1}H NMR spectra of the different studied mixtures of System 2: ACN + DX obtained by the ^{1}H NMR and ^{1}H NMR ODNP experiments in continuous-flow. Two singlet peaks can be identified that are assigned to ACN and DX. By switching on the MW and performing the ^{1}H NMR ODNP experiment, a significant improvement in the SNR is achieved. The average signal enhancements are $E_{\mathrm{ACN}}^{^1\mathrm{H}} = 6$ for ACN and $E_{\mathrm{DX}}^{^1\mathrm{H}} = 6$ for DX for System 2.

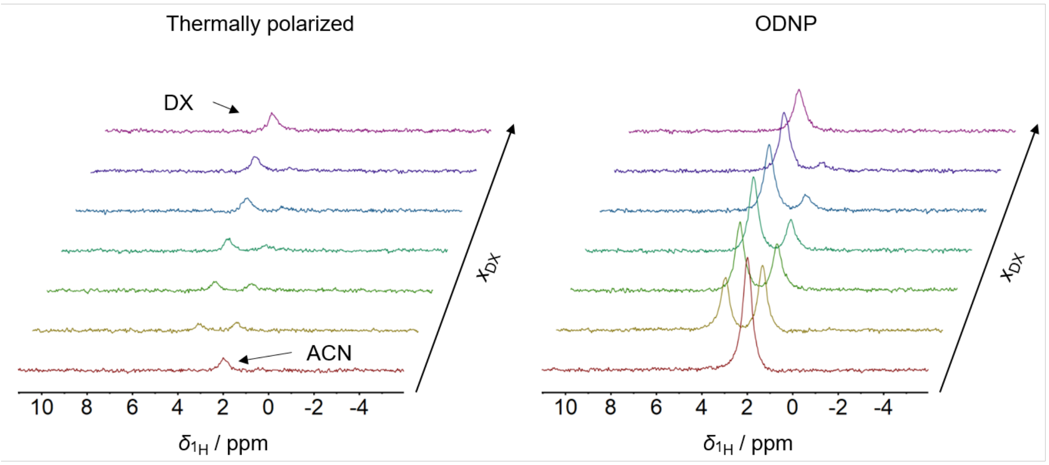

Figure SI.4: ^{1}H NMR spectra of System 2: ACN + DX for mixtures with different composition acquired with a single scan in continuous-flow (flow velocity v = 0.34 m s^{-1}). Left: thermal experiments. Right: ODNP experiments.

In Figure SI.5, the obtained signal integrals of ACN and DX of System 2, which are acquired with the ^{1}H NMR ODNP experiment, are shown as a function of the composition. The corresponding signal enhancements are displayed in Figure SI.6. The relative deviations of the data points from the calibration curve are shown in Figure SI.7. Table SI.3 provides the numerical values for the signal integrals and the uncorrected mole fractions x_i^{ODNP} acquired with the ^{1}H NMR ODNP experiment as well as the spin-lattice relaxation time $T_{1,\,^1\mathrm{H}}$ of ACN and DX in the mixtures.

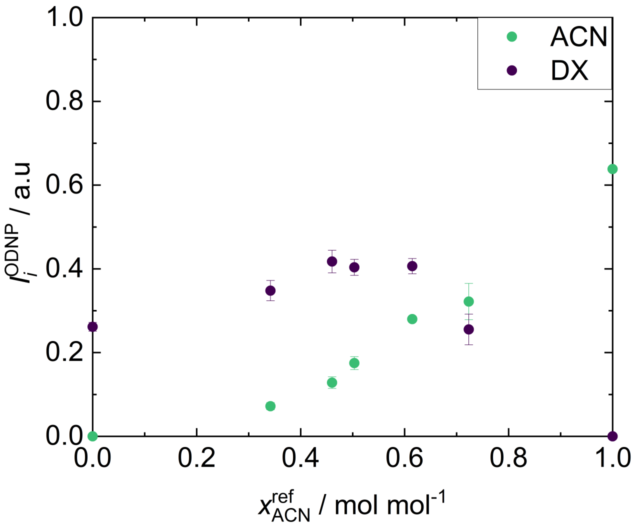

Figure SI.5: Signal integrals of ACN and DX of System 2 as a function of the sample's composition acquired with the ^{1}H NMR ODNP experiment.

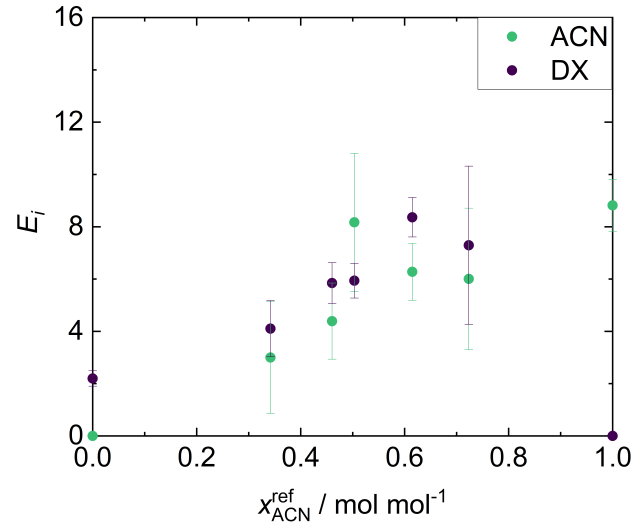

Figure SI.6: Signal enhancements of ACN and DX of System 2 as a function of the sample's composition acquired with the ^{1}H NMR ODNP experiment.

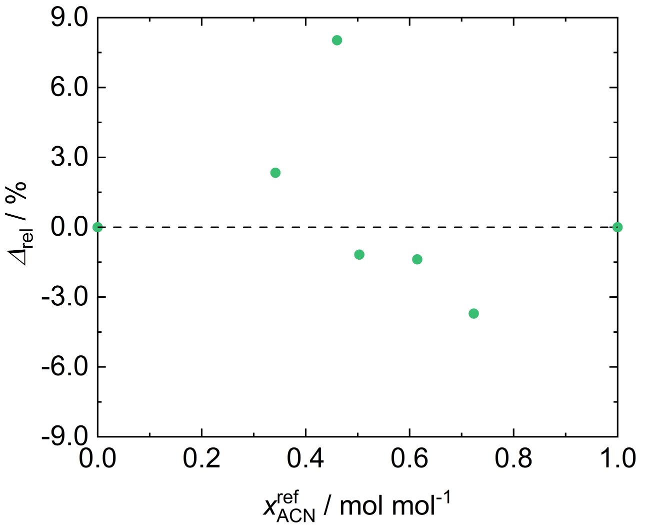

Figure SI.7: Relative deviations of the data points obtained with the ^{1}H NMR ODNP experiment from the calibration curve of System 2: ACN + DX.

Table SI.3: Measured signal integrals I^{ODNP} of ACN and DX, their signal enhancements $E^{^1\mathrm{H}}$ and the uncorrected mole fraction x_i^{ODNP} as a function of the composition acquired with the ^{1}H NMR ODNP experiment. Spin-lattice relaxation time $T_{1,\,^1\mathrm{H}}$ at a magnetic field strength of 1 T of selected mixtures are additionally given. The ^{1}H NMR ODNP experiments were repeated 5 times; $T_{1,\,^1\mathrm{H}}$ were obtained with 3 repetitions. Standard uncertainties and errors are given, respectively.

Mixture	ACN				DX		
x_{ACN} / mol mol^{-1}	I^{ODNP} / a.u.	$E^{^1\mathrm{H}}$	x^{ODNP} / mol mol^{-1}	$T_{1,\,^1\mathrm{H}}$ / s	I^{ODNP} / a.u.	$E^{^1\mathrm{H}}$	$T_{1,\,^1\mathrm{H}}$ / s
0	-	-	0	-	0.26±0.01	2.2±0.3	3.58±0.01
0.342	0.07±0.01	3.0±2.1	0.355±0.017	3.16±0.01	0.35±0.02	4.1±1.1	2.87±0.01
0.461	0.13±0.01	4.4±1.5	0.450±0.037	-	0.42±0.03	5.8±0.8	-
0.503	0.17±0.01	8.2±2.6	0.535±0.018	3.38±0.01	0.40±0.02	5.9±0.7	3.12±0.01
0.615	0.28±0.01	6.3±1.1	0.648±0.014	-	0.41±0.02	8.4±0.8	-
0.724	0.32±0.04	6.0±2.7	0.771±0.007	3.61±0.01	0.26±0.04	7.3±3.0	3.38±0.02
1	0.64±0.01	8.8±1.0	1	3.99±0.01	-	-	-

System 3: Acetonitrile (ACN) + Chloroform (CF)

Figure SI.8 displays the ^{1}H NMR spectra of the different studied mixtures of System 3: ACN + CF obtained by the ^{1}H NMR and ^{1}H NMR ODNP experiments in continuous-flow. Two singlet peaks can be identified that are assigned to ACN and CF. By switching on the MW and performing the ^{1}H NMR ODNP experiment, a significant improvement in the SNR is achieved. The average signal enhancements are $E_{\mathrm{ACN}}^{^1\mathrm{H}} = 5$ for ACN and $E_{\mathrm{DX}}^{^1\mathrm{H}} = 7$ for CF for System 3.

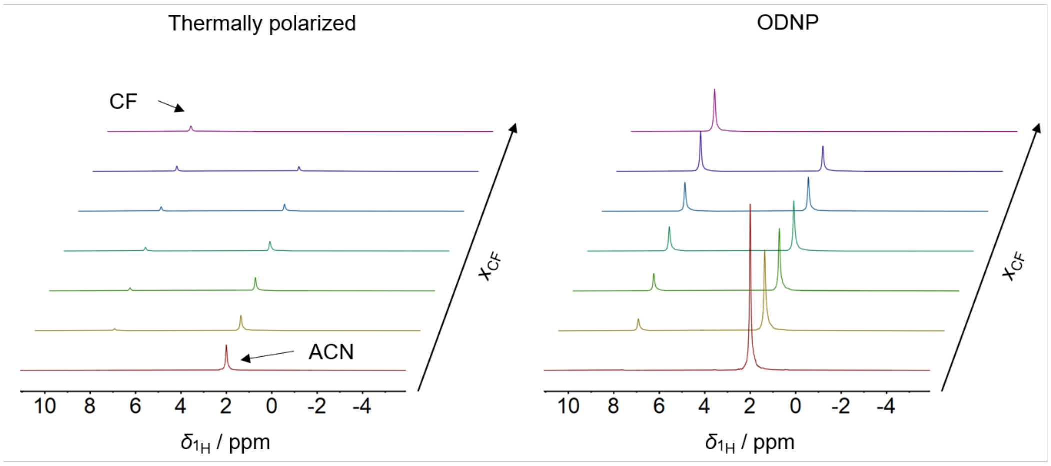

Figure SI.8: ^{1}H NMR spectra of System 3: ACN + CF for mixtures with different composition acquired with a single scan in continuous-flow (flow velocity v = 2.38 m s^{-1}). Left: thermal experiments. Right: ODNP experiments.

In Figure SI.9, the obtained signal integrals of ACN and CF, which are acquired with the ^{1}H NMR ODNP experiment, are shown as a function of the composition. The corresponding signal enhancements are displayed in Figure SI.10. The relative deviations of the data points from the calibration curve are shown in Figure SI.11. Table SI.4 provides the numerical values for the signal integrals and the uncorrected mole fractions x_i^{ODNP} acquired with the ^{1}H NMR ODNP experiment as well as the spin-lattice relaxation time $T_{1,^1\mathrm{H}}$ of ACN and CF in the mixtures.

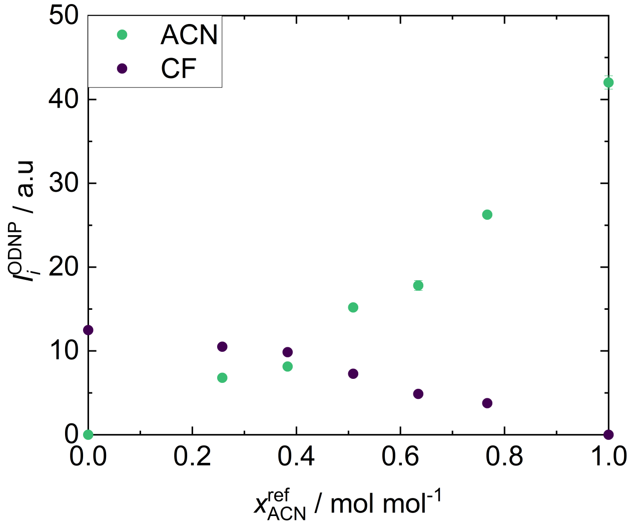

Figure SI.9: Signal integrals of ACN and CF of System 3 as a function of the sample's composition acquired with the ^{1}H NMR ODNP experiment.

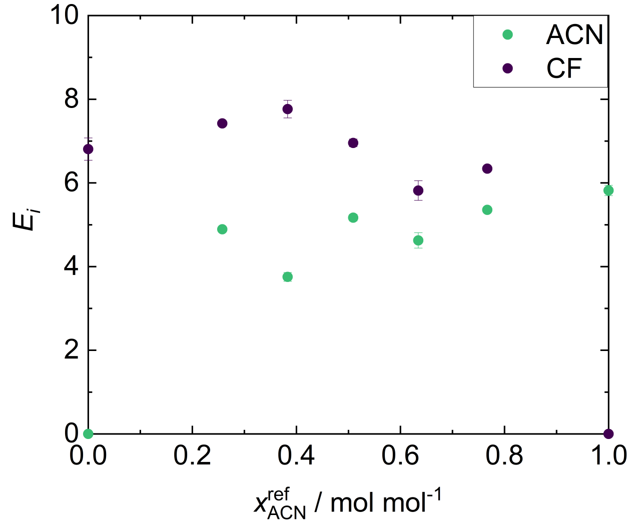

Figure SI.10: Signal enhancements of ACN and CF of System 3 as a function of the sample's composition acquired with the ^{1}H NMR ODNP experiment.

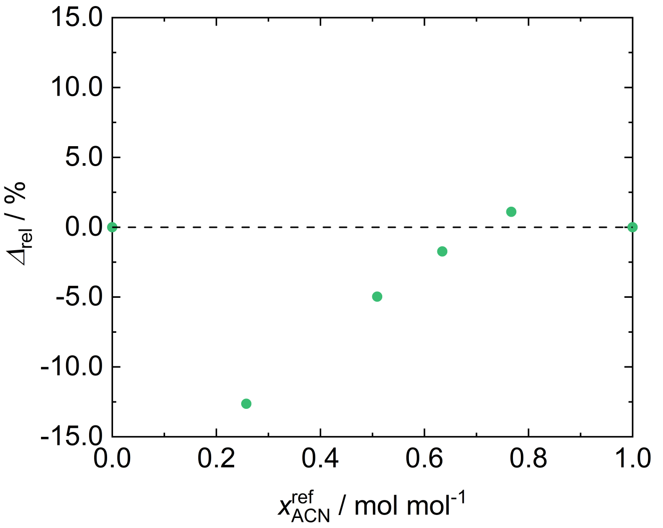

Figure SI.11: Relative deviations of the data points obtained with the ^{1}H NMR ODNP experiment from the calibration curve of System 3: ACN + CF.

Table SI.4: Measured signal integrals I^{ODNP} of ACN and CF, their signal enhancements $E^{^1\mathrm{H}}$ and the uncorrected mole fraction x_i^{ODNP} as a function of the composition acquired with the ^{1}H NMR ODNP experiment. Spin-lattice relaxation time $T_{1,\,^1\mathrm{H}}$ at a magnetic field strength of 1 T of selected mixtures are additionally given. The ^{1}H NMR ODNP experiments were repeated 5 times; $T_{1,\,^1\mathrm{H}}$ were obtained with 3 repetitions. Standard uncertainties and errors are given, respectively.

Mixture	ACN				CF		
x_{ACN} / mol mol^{-1}	I^{ODNP} / a.u.	$E^{^1\mathrm{H}}$	x^{ODNP} / mol mol^{-1}	$T_{1,\,^1\mathrm{H}}$ / s	I^{ODNP} / a.u.	$E^{^1\mathrm{H}}$	$T_{1,\,^1\mathrm{H}}$ / s
0	-	-	0	-	12.49±0.31	6.8±0.3	5.25±0.06
0.258	6.79±0.06	4.9±0.1	0.177±0.001	3.59±0.05	10.51±0.08	7.4±0.1	4.41±0.04
0.383	8.13±0.41	3.8±0.1	0.216±0.001	-	9.84±0.34	7.8±0.2	-
0.509	15.18±0.16	5.2±0.1	0.410±0.001	3.23±0.05	7.27±0.07	7.0±0.1	4.03±0.04
0.634	17.81±0.88	4.6±0.2	0.549±0.001	-	4.87±0.24	5.8±0.2	-
0.767	26.26±0.26	5.4±0.1	0.699±0.001	3.58±0.05	3.77±0.03	6.3±0.1	4.41±0.04
1	42.01±0.79	5.8±0.1	1	3.99±0.01	-	-	-

In Figure SI.12, the obtained signal integrals of ACN and CF, which are acquired with the ^{13}C NMR ODNP experiment, are shown as a function of the composition. The corresponding signal enhancements are displayed in Figure SI.13. The relative deviations of the data points from the calibration curve are shown in Figure SI.14. Table SI.4 provides the numerical values of the signal integrals and the uncorrected mole fractions x_i^{ODNP} acquired with the ^{13}C NMR ODNP experiment.

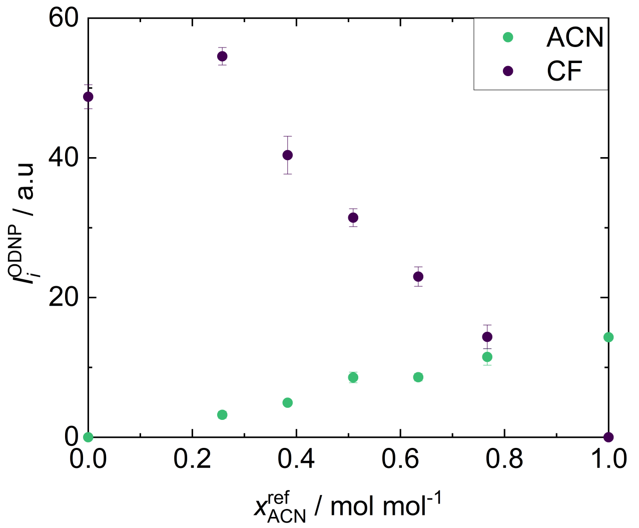

Figure SI.12: Signal integrals of ACN and CF of System 3 as a function of the mixtures's composition acquired with the ^{13}C NMR ODNP experiment.

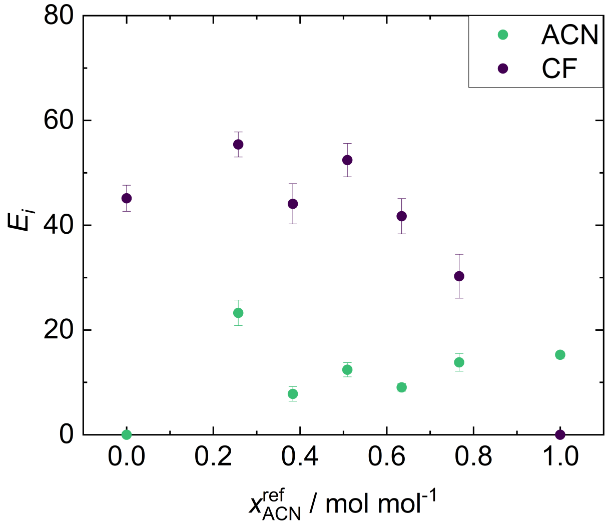

Figure SI.13: Signal enhancements of ACN and CF of System 3 as a function of the mixtures's composition acquired with the ^{13}C NMR ODNP experiment.

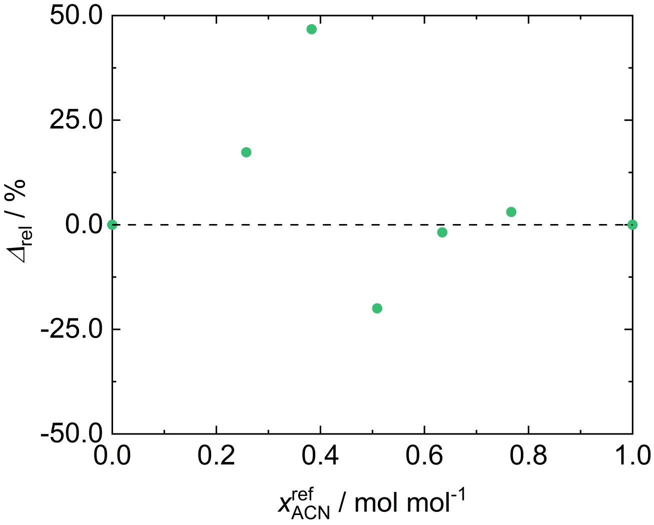

Figure SI.14: Relative deviations of the data points obtained with the ^{13}C NMR ODNP experiment from the calibration curve of System 3: ACN + CF.

Table SI.5: Measured signal integrals I^{ODNP} of ACN and CF, their signal enhancements $E^{^{13}\mathrm{C}}$ and the uncorrected mole fraction x_i^{ODNP} as a function of the composition acquired with the ^{13}C NMR ODNP experiment. The ^{13}C NMR ODNP experiments were repeated 3 times. Standard uncertainties and errors are given, respectively.

Mixture	ACN			CF	
x_{ACN} / mol mol^{-1}	I^{ODNP} / a.u.	$E^{^{13}\mathrm{C}}$	x^{ODNP} / mol mol^{-1}	I^{ODNP} / a.u.	$E^{^{13}\mathrm{C}}$
0	-	-	0	48.74±1.71	45.1±2.5
0.258	3.21±0.52	23.3±2.4	0.074±0.009	54.53±1.25	55.4±2.4
0.383	4.95±0.42	7.8±1.4	0.083±0.005	40.38±2.71	44.1±3.8
0.509	8.56±0.76	12.4±1.4	0.214±0.014	31.43±1.28	52.4±3.2
0.634	8.59±0.52	9.0±0.7	0.272±0.006	23.00±1.39	41.7±3.4
0.767	11.50±1.19	13.8±1.7	0.445±0.041	14.37±1.70	30.2±4.2
1	14.32±0.35	15.3±0.7	1	-	-

References

(1) Kircher, R.; Hasse, H.; Münnemann, K. High Flow-Rate Benchtop NMR Spectroscopy Enabled by Continuous Overhauser DNP. *Analytical Chemistry* **2021**, *93*, 8897–8905.

(2) Phuong, J.; Salgado, B.; Labusch, T.; Hasse, H.; Münnemann, K. Overhauser Dynamic Nuclear Polarization Enables Single Scan Benchtop ^{13}C NMR Spectroscopy in Continuous-Flow. *in revision at Analytical Chemistry* **2024**,
